# The FemTech revolution: Unlocking the potential of new technology for optimizing pregnancy outcomes in low‐ and middle‐income countries and remote areas

**DOI:** 10.1002/ijgo.71156

**Published:** 2026-06-19

**Authors:** Nir Melamed, Hema Divakar, Mahesh Choolani, Fionnuala M. McAuliffe, Lin Foo, Hassan Shehata, Gabriel Jones, Vyta Senikas, Eline M. Van der Beek, Nandita Palshetkar, Gabriele Saccone, Vincenzo Berghella, Mauricio A. Cuello, Justin Konje, Bhaskar Bhatt, Augusto Cam, Moses Obimbo, Aris Papageorghiou, Moshe Hod, the FIGO Committee on Women's Health and Technology

**Affiliations:** ^1^ Division of Maternal Fetal Medicine, Department of Obstetrics and Gynaecology, Sunnybrook Health Sciences Centre University of Toronto Toronto Ontario Canada; ^2^ Divakars Speciality Hospital Bengaluru India; ^3^ Department of Obstetrics and Gynaecology, Yong Loo Lin School of Medicine National University of Singapore Singapore Singapore; ^4^ Department of Obstetrics and Gynaecology, National University Centre for Women and Children National University Health System Singapore Singapore; ^5^ UCD Perinatal Research Centre, School of Medicine University College Dublin Dublin Ireland; ^6^ National Maternity Hospital Dublin Ireland; ^7^ Department of Obstetrics & Gynaecology National University Hospital Singapore Singapore; ^8^ Centre for Reproductive Immunology and Pregnancy St. George's, Epsom and St. Helier Group London UK; ^9^ Oxford Digital Health Labs, Nuffield Department of Women's and Reproductive Health University of Oxford Oxford UK; ^10^ Faculty of Medicine McGill University Montreal Québec Canada; ^11^ Department of Paediatrics, University Medical Centre Groningen University of Groningen Groningen the Netherlands; ^12^ Nestle Institute of Health Sciences, Nestle Research Lausanne Switzerland; ^13^ Lilavati Hospital Mumbai India; ^14^ Department of Neuroscience, Reproductive Science and Dentistry, School of Medicine University of Naples Federico II Naples Italy; ^15^ Department of Maternal‐Fetal Medicine, Department of Obstetrics and Gynecology Thomas Jefferson University Philadelphia Pennsylvania USA; ^16^ Division of Obstetrics and Gynecology, School of Medicine Pontificia Universidad Católica de Chile Santiago Chile; ^17^ Feto‐Maternal Centre Doha Qatar; ^18^ Department of Obstetrics & Gynaecology Weill Cornell Medicine Doha Qatar; ^19^ School of Design UPES Dehradun India; ^20^ Universidad Peruana de Ciencias Aplicadas Lima Peru; ^21^ Department of Human Anatomy & Medical Physiology University of Nairobi Nairobi Kenya; ^22^ Nuffield Department of Women's & Reproductive Health Oxford Maternal and Perinatal Health Institute (OMPHI), University of Oxford Oxford UK; ^23^ Mor Comprehensive Women's Health Care Center, Sackler Faculty of Medicine Tel Aviv University Tel‐Aviv Israel

**Keywords:** framework, LMIC, pregnancy, technology

## Abstract

Maternal and neonatal mortality and morbidity rates uncover major global health disparities. Despite ongoing efforts, the rates of maternal and neonatal complications remain substantially higher in low‐ and middle‐income countries (LMICs) compared to high‐income countries (HICs). These high rates are the result of several unmet needs in LMICs, including limited access to quality antenatal care, health worker shortages, unreliable infrastructure, sociocultural barriers, low health literacy, environmental and nutritional challenges, and affordability. In addition, while the greatest burden of these complications lies in LMICs, it is crucial to recognize that similar disparities exist in rural and remote areas of large, higher‐income countries. FemTech (female technology), which refers to a wide range of digital tools and technologies designed specifically to support women's health, has the potential to address these unmet needs in LMICs. In many LMIC settings, mobile connectivity may represent the most scalable digital infrastructure available to women, often reaching communities long before formal health system expansion. However, the uptake of these in LMICs remains limited by infrastructure, regulatory, affordability, and sociocultural constraints. Introducing these digital solutions to LMICs without careful adaptations to these unique factors is more likely to widen rather than narrow inequities. Many international guidelines advocating the implementation of advanced technologies have not taken into account these unique LMIC‐specific challenges. This gap underscores the need to develop strategies for the implementation of FemTech in LMIC settings. FIGO and its partners are well placed to coordinate the development of dedicated global guidance tailored to resource‐limited settings. This document is a first step toward this goal.

## 1 EXECUTIVE SUMMARY

### 1.1 Background

Maternal and neonatal mortality and morbidity rates uncover major global health disparities. Despite ongoing efforts, the rates of maternal and neonatal complications remain substantially higher in low‐ and middle‐income countries (LMICs) compared to high‐income countries (HICs). These high rates are the result of several unmet needs in LMICs, including limited access to quality antenatal care, health worker shortages, unreliable infrastructure, sociocultural barriers, low health literacy, environmental and nutritional challenges, and affordability. In addition, although the greatest burden of these complications lies in LMICs, it is crucial to recognize that similar disparities exist in rural and remote areas of large, higher‐income countries.

**FIGURE 1:**
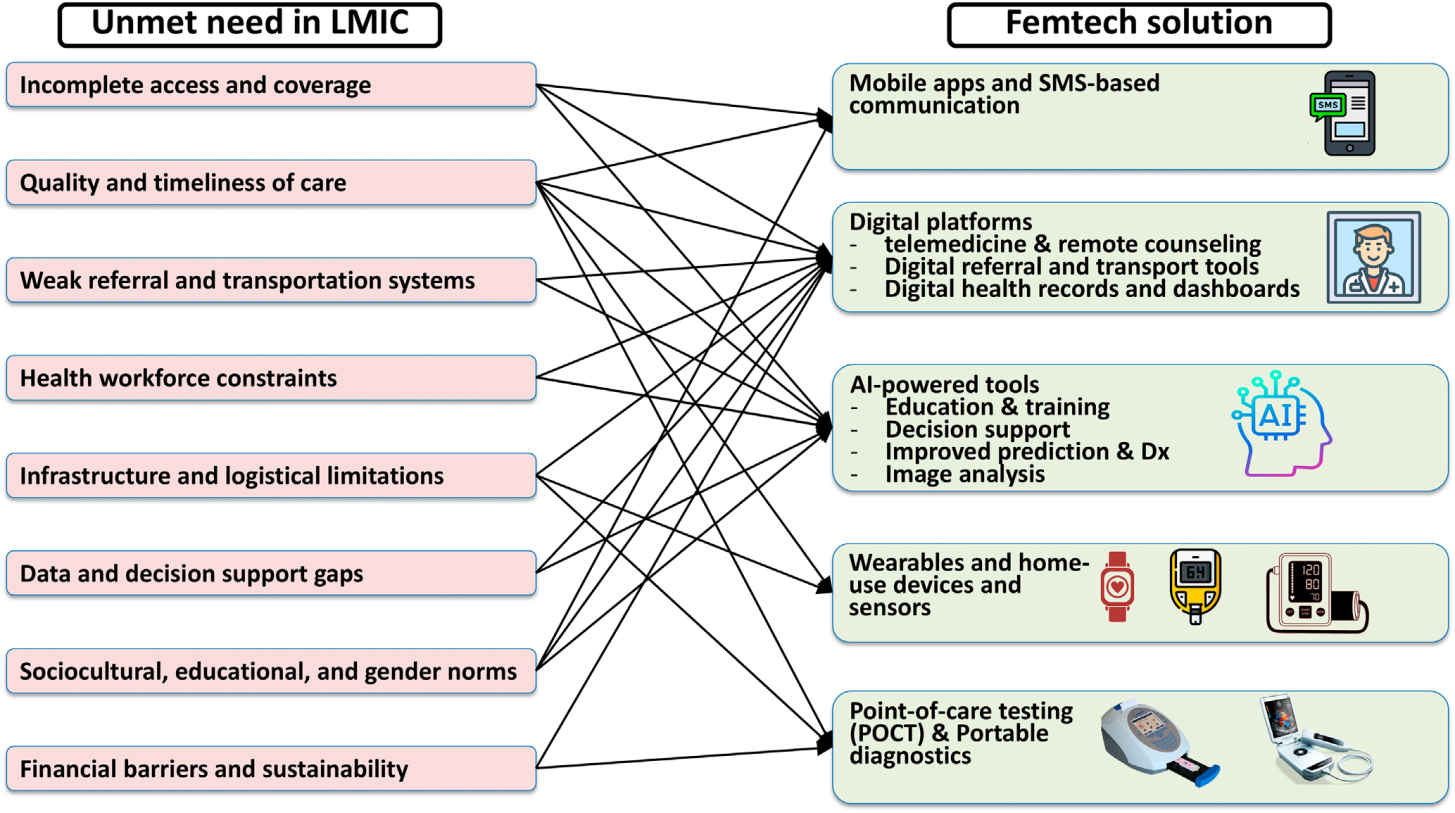
The potential of FemTech in addressing LMICs' unmet needs. The figure illustrates the mapping of key unmet needs to the corresponding FemTech solution categories. AI, artificial intelligence; LMIC, low‐ and middle‐income country.

FemTech (female technology), which refers to a wide range of digital tools and technologies designed specifically to support women's health, has the potential to address these unmet needs in LMICs (Table 1, Figure 1). In many LMIC settings, mobile connectivity may represent the most scalable digital infrastructure available to women, often reaching communities long before formal health system expansion. However, the uptake of these in LMICs remains limited by infrastructure, regulatory, affordability, and sociocultural constraints. Introducing these digital solutions to LMICs without careful adaptations to these unique factors is more likely to widen rather than narrow inequities. Many international guidelines advocating the implementation of advanced technologies have not taken into account these unique LMIC‐specific challenges. This gap underscores the need to develop strategies for the implementation of FemTech in LMIC settings. The International Federation of Gynecology and Obstetrics (FIGO) and its partners are well placed to coordinate the development of dedicated global guidance tailored to resource‐limited settings. This document is a first step toward this goal.

**TABLE 1:** Addressing LMICs' unmet needs through FemTech and advanced technologies.

Unmet need in LMICs	Potential FemTech/advanced technology solutions
Incomplete access and coverage	Mobile apps and SMS for ANC reminders, maternal education, and local language content Tele‐ANC hybrid models offering remote consultation and triage mHealth tools to guide self‐monitoring and promote ANC attendance
Quality and timeliness of care	AI‐assisted decision support for community health workers and midwives Home‐use wearable devices (e.g., BP cuffs, fetal heart monitors, uterine contraction sensors, home ultrasound) with telemonitoring centralized ANC models combining frontline care with expert oversight AI‐based risk scoring tools Portable diagnostic devices (e.g., handheld ultrasound) Remote consultation platforms for timely escalation of care
Poorer referral and transportation systems	Digital communication platforms for referral coordination Real‐time communication and data sharing tools with higher‐level facilities Mobile platforms for transport alerts and planning AI risk stratification to prioritize referrals GPS‐enabled emergency transport apps Community‐based digital birth preparedness checklists
Health workforce constraints	Telemedicine and tele‐mentoring platforms AI‐driven clinical protocol and decision support tools Digital tools to support task‐shifting to non‐physician providers E‐learning modules and AI‐powered virtual simulations for ongoing provider training
Infrastructure and logistical limitations	Home‐based and portable POCTs for anemia (Hb), infections (HIV, syphilis), and preterm birth (fetal fibronectin) Wearables and solar‐powered devices Cloud‐based systems with offline functionality Mobile diagnostic kits requiring minimal infrastructure (e.g., handheld ultrasound) Digital inventory management tools to improve supply chains
Data and decision support gaps	AI‐driven analytics for real‐time risk prediction and triage, integrating remote monitoring data from wearables Digital dashboards for health system monitoring, quality improvement, real‐time surveillance, and early warning Mobile data collection tools Digital maternal and child health records
Sociocultural, educational, and gender norms	Culturally adapted mobile apps and digital education platforms in local languages with co‐creation with end users Audio/video storytelling to improve health literacy Private digital mental health tools for stigma‐sensitive care Adolescent‐ and family‐focused content to support informed decision‐making Anonymous online counseling for adolescents Tools to engage male partners and families
Financial barriers and sustainability	Low‐cost mobile and POCT solutions Digital insurance and micro‐financing platforms for maternal care Telehealth services to reduce out‐of‐pocket costs AI‐powered planning tools for governments to optimize resource allocation Digital payment systems for transport or medications Public‐private partnerships to support scale‐up and long‐term sustainability

Abbreviations: AI, artificial intelligence; ANC, antenatal care; Hb, hemoglobin; LMIC, low‐ and middle‐income country; POCT, point‐of‐care testing.

### 1.2 Purpose of this document

In this document, we review the evidence on the benefits of FemTech for improving antenatal care and pregnancy outcomes, describe the main barriers to implementing FemTech in LMICs, propose FIGO‐aligned adaptations and strategies to address these barriers, and outline a practical framework to guide implementation. This document should be viewed as a strategic guidance paper, developed under the direction of the FIGO Committee on Women’s Health and Technology, rather than a formal systematic review or clinical practice guideline.

### 1.3 Target audience

This guideline is directed at multiple stakeholders, including healthcare providers and educators, health system leaders and policymakers, regulators and governance bodies, professional and academic organizations, and technology and industry partners.

### 1.4 FemTech for pregnancy care: Evidence of benefits and outcomes

Pregnancy‐related FemTech solutions can be classified into five broad categories: (1) mobile apps and SMS‐based communication tools for patient education, health tracking, reminders, and communication; (2) wearables, home‐use devices, monitors, and sensors for monitoring of blood pressure (BP), heart rate, glucose, maternal weight, fetal heart rate, and uterine contractions; (3) point‐of‐care testing (POCT) devices, such as handheld ultrasound, and tests for conditions such as anemia, HIV, syphilis, preterm birth risk, and pre‐eclampsia; (4) artificial intelligence (AI)‐powered tools for education and training, decision support, risk stratification, improved dynamic prediction and diagnosis, and automated image analysis; and (5) digital platforms, such as telemedicine and remote counseling, which support centralized antenatal care models with trained providers and AI‐based decision systems.

Evidence across all the FemTech categories supports their integration into maternal and perinatal care (Table 2). In addition, wearables and home‐use devices offer the potential for individual‐level experimentation (n‐of‐1 studies), a research design that focuses on serial measurements from a single individual over time, thereby laying the foundation for personalized diagnostic and therapeutic interventions in pregnancy.

**TABLE 2:** FemTech for pregnancy care: evidence of benefits.

Category	Use case	Individual products	Evidence summary	Caveats
Mobile apps SMS‐based communication	Health education and danger signs Reminders Care engagement and navigation Pregnancy self‐management Bidirectional communication with care providers		Improves: ANC and skilled birth attendance Physical and mental health. GDM Apps: improved glycemic control, reduced insulin needs, lower gestational weight gain, and macrosomia Immunization uptake Breastfeeding	Impact depends on: Implementation fidelity Language and literacy fit Handset and data access Privacy and consent Integration with health information systems
Wearables, home‐use devices, and sensors	Reduce clinic visits Home monitoring of high‐risk pregnancies (diabetes, hypertension, growth restriction, preterm birth)	Home BP cuffs with telemonitoring	Safe, but did not accelerate detection without responsive pathways Cost‐effectiveness depends on workflow	Requires linkage to treatment protocols and escalation pathways Device quality, connectivity, maintenance, and training matter Regulatory clearance varies Equity risks if devices are user‐paid
		Bluetooth glucometers CGM and hybrid closed‐loop systems Home OGTT	Improves control Reduce pre‐eclampsia, neonatal hypoglycemia, and macrosomia Improved screening uptake and accuracy	
		Home FHR devices	High agreement with standard monitoring	
		Tocometers		
		Connected scales	Reduce gestational weight gain	
		Home ultrasound	High satisfaction and feasible remote assessment	
POCT for home or local facilities	Rapid screening for infectious disease, anemia	HIV/syphilis rapid testing	Increase same‐day diagnosis and treatment Endorsed by WHO Improve coverage Save costs	Quality control, supply chains, and environmental conditions affect accuracy Needs confirmatory pathways, staff training, and data capture Cost and procurement models must be planned Integration with referral and treatment is essential
		Hemoglobin	Widely used, useful triage Accuracy varies based on settings	
	Triage of patients with suspected preterm labor and pre‐eclampsia	Fetal fibronectin	High negative predictive value Reduces unnecessary admissions and transfers	
		sFlt‐1 and PlGF assays	sFlt‐1/PlGF improves risk stratification and time‐to‐diagnosis and can avoid unnecessary interventions	
	Assessment of fetal growth and well‐being by minimally trained users	Handheld ultrasound with optional AI pairing	Handheld ultrasound can achieve clinically acceptable biometry; trained midwife programs show high accuracy and faster turnaround	
AI‐powered tools	Education and simulation	AI conversational tools and adaptive learning		Must meet WHO AI guidance on validation, transparency, safety, and post‐market surveillance Risks include bias, inequitable performance, data protection, and generalizability limits Requires governance, clinician oversight, and reliable devices
	Risk prediction and early diagnosis	Predictive models from routine data and wearables		
	Guideline adherence	Digital decision support	Improves ANC quality	
	Assessment of gestational age and anomalies detection	Automated image analysis for blind‐sweep ultrasound	Can estimate gestational age from blind sweeps with accuracy comparable to trained sonographers and automate basic biometry and anomaly detection	
Digital platforms: telemedicine, remote counseling, centralized ANC models	Combine virtual contacts with targeted in‐person examinations, imaging, and labs Centralized review and referral Remote data review and counseling	Hybrid tele‐ANC frameworks Telemedicine support for care providers	Improved access and quality of ANC Increased satisfaction and reduced stress Improved adherence and clinical outcomes Fewer clinic visits Improve ANC content and quality Reduce documentation burden	Connectivity Device access Staffing and reimbursement Legal and safeguarding frameworks and reliable referral pathways are prerequisites Careful monitoring for rare outcomes is needed at scale Integration with national data systems is critical

Abbreviations: AI, artificial intelligence; ANC, antenatal care; BP, blood pressure; CGM, continuous glucose monitoring; FHR, fetal heart rate; GDM, gestational diabetes mellitus; LMIC, low‐ and middle‐income country; OGTT, oral glucose tolerance test; PlGF, placental growth factor; POCT, point‐of‐care testing; sFlt‐1, soluble fms‐like tyrosine kinase 1.

**FIGURE 2:**
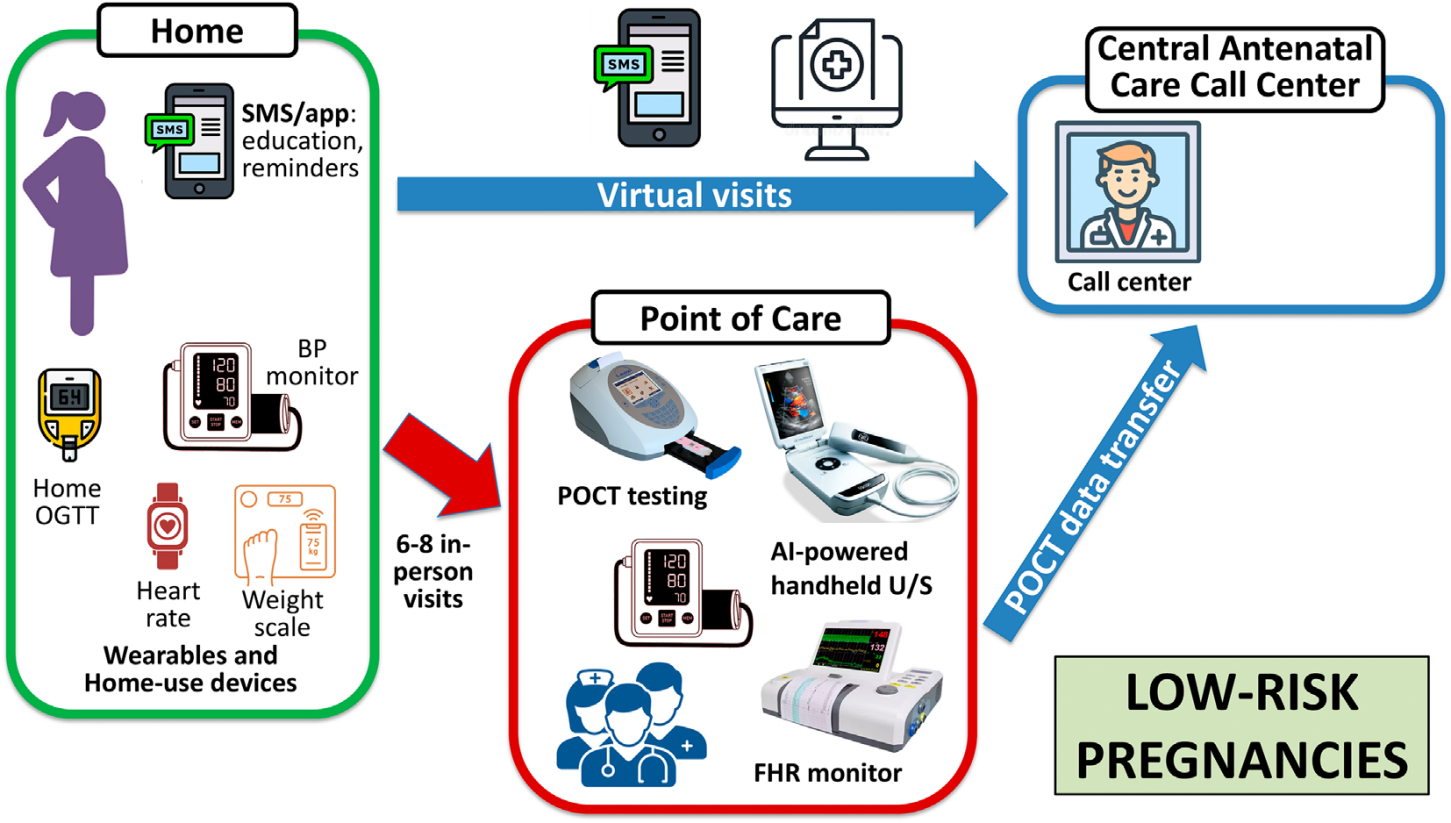
A care model illustrating the use of FemTech for the care of low‐risk pregnancies. In low‐risk pregnancies, the main role of FemTech is to increase access to routine ANC, reduce the logistical and financial burden on families and health systems, risk stratification, and ensure timely detection of complications. Mobile apps and SMS reminders improve adherence to recommended antenatal schedules, while digital educational platforms enhance maternal knowledge and encourage healthy behaviors such as adequate nutrition and recognition of danger signs. Wearables and simple home‐use devices enable women to monitor their health with remote oversight from care providers. Hybrid tele‐ANC models can reduce travel costs and improve patient satisfaction, particularly in rural or underserved areas. These strategies promote equitable access to essential care for low‐risk populations. AI, artificial intelligence; ANC, antenatal care; BP, blood pressure; FHR, fetal heart rate; OGTT, oral glucose tolerance test; POCT, point‐of‐care testing; U/S, ultrasound.

**FIGURE 3:**
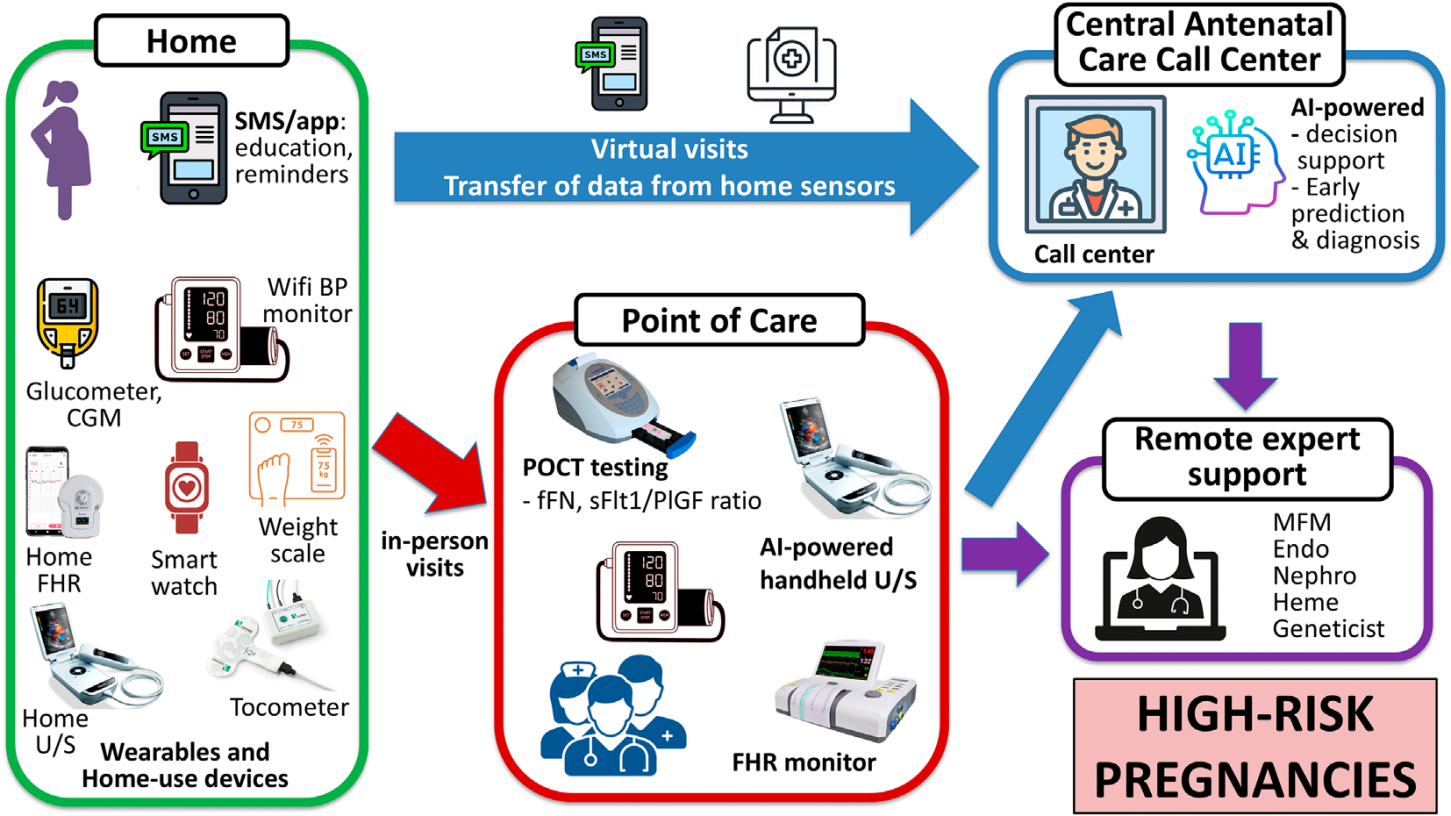
A care model illustrating the use of FemTech for the care of high‐risk and complicated pregnancies. For women with high‐risk pregnancies, continuous or frequent monitoring with wearables enables early detection of deterioration and allows timely adjustment of treatments. AI‐powered risk stratification tools can process continuous data streams to trigger alerts and guide escalation to higher levels of care. Point‐of‐care diagnostics such as portable ultrasound or rapid tests for pre‐eclampsia biomarkers and fetal fibronectin for preterm birth can be used at local, peripheral health centers or even at home, avoiding unnecessary transfers while ensuring timely diagnosis and referral. Telemedicine and centralized care platforms that connect frontline providers with specialists can facilitate timely support in managing complex cases, thereby increasing access to high‐quality care. AI, artificial intelligence; BP, blood pressure; CGM, continuous glucose monitoring; Endo, endocrinology; FHR, fetal heart rate; Heme, hematology; MFM, maternal‐fetal medicine; Nephro, nephrology; OGTT, oral glucose tolerance test; POCT, point‐of‐care testing; U/S, ultrasound.

The most compelling benefits are likely to be achieved when these technologies are combined. Together, they can improve access to antenatal care, with potential to enhance care quality, detect early warning signals, bring diagnostics closer to patients, standardize decision‐making, and streamline the escalation of care in both low‐risk (Figure 2) and high‐risk pregnancies (Figure 3).

### 1.5 Challenges, barriers, and proposed adaptations to implementing FemTech in LMICs

The potential benefits of FemTech to LMICs will only be achieved if innovations are adapted to their unique needs, including unreliable infrastructure (e.g., power, Internet connectivity, and device maintenance); human factors (e.g., digital literacy, provider workload, and human acceptance); policy, regulation, and legal and ethical barriers; sociocultural factors and equity; and cost, affordability, and sustainability. We propose a range of strategies and adaptations to overcome each of these barriers. These strategies, which are described in detail in Section 5 and summarized in Table 3, range from solar power and offline‐capable tools for infrastructure constraints, to capacity‐building and task‐shifting for digital literacy challenges, privacy‐by‐design for regulatory gaps, community co‐creation for sociocultural barriers, and bulk purchasing with public‐private partnerships for cost and sustainability.

**TABLE 3:** Challenges and barriers to implementing FemTech in low‐ and middle‐income countries.

Barrier/challenge	Examples	Proposed adaptations and strategies
Infrastructure, connectivity constraints, and device maintenance	Intermittent/no electricity Poor mobile/broadband Device maintenance and durability Fragmented/non‐standardized health information systems and poor interoperability	Solar/battery backup Solar panel installations Hybrid connectivity (offline caching, SMS fallback) Local device adaptation Durable hardware, spare‐parts planning, local repair networks Community Internet hubs/shared digital commons Adopt interoperability standards
Digital literacy, provider workload, and human acceptance	Low digital literacy among women and health team Usability and language mismatches Cultural resistance/distrust, overburdened staff, and workflow disruption	Comprehensive capacity‐building Training and mentorship User‐friendly manuals/interfaces Task‐shifting to less‐specialized workers Ensure leadership engagement Digital solutions should minimize extra steps, be embedded into workflows, and automate data capture
Policy, regulation, and legal and ethical barriers	Data privacy and weak/absent data‐protection laws and oversight Policy gaps for device approval Lack of telemedicine reimbursement Slow regulatory adaptation	Sustained policy advocacy and legal framework development Ensure data privacy/security even in low‐connectivity settings through privacy‐by‐design and encryption Implement ethical AI/digital health guardrails
Sociocultural factors and equity	Women in remote and low‐income groups lack smartphones/data plans/digital literacy Gender norms restrict phone access/autonomy Language barriers and low health literacy	Community engagement, local champions Culturally sensitive design Awareness campaigns via social media for norm change Prioritize rural/marginalized groups Close the gendered usage gap via device subsidies, community access points, data‐lite designs, safety features Equity monitoring (sex/age/geography/wealth)
Cost, affordability, and sustainability	High upfront and recurrent costs Uncertain revenue/reimbursement Donor fatigue Poor alignment with national priorities Heavy out‐of‐pocket burden and limited insurance coverage	Ensure sustainable funding and policy alignment Employ innovative financing: bulk purchasing, subsidized pricing Promote local manufacturing Emphasize stakeholder engagement and long‐term viability planning

Abbreviation: AI, artificial intelligence.

### 1.6 Getting it right: A practical framework for implementing FemTech in LMICs

**FIGURE 4:**
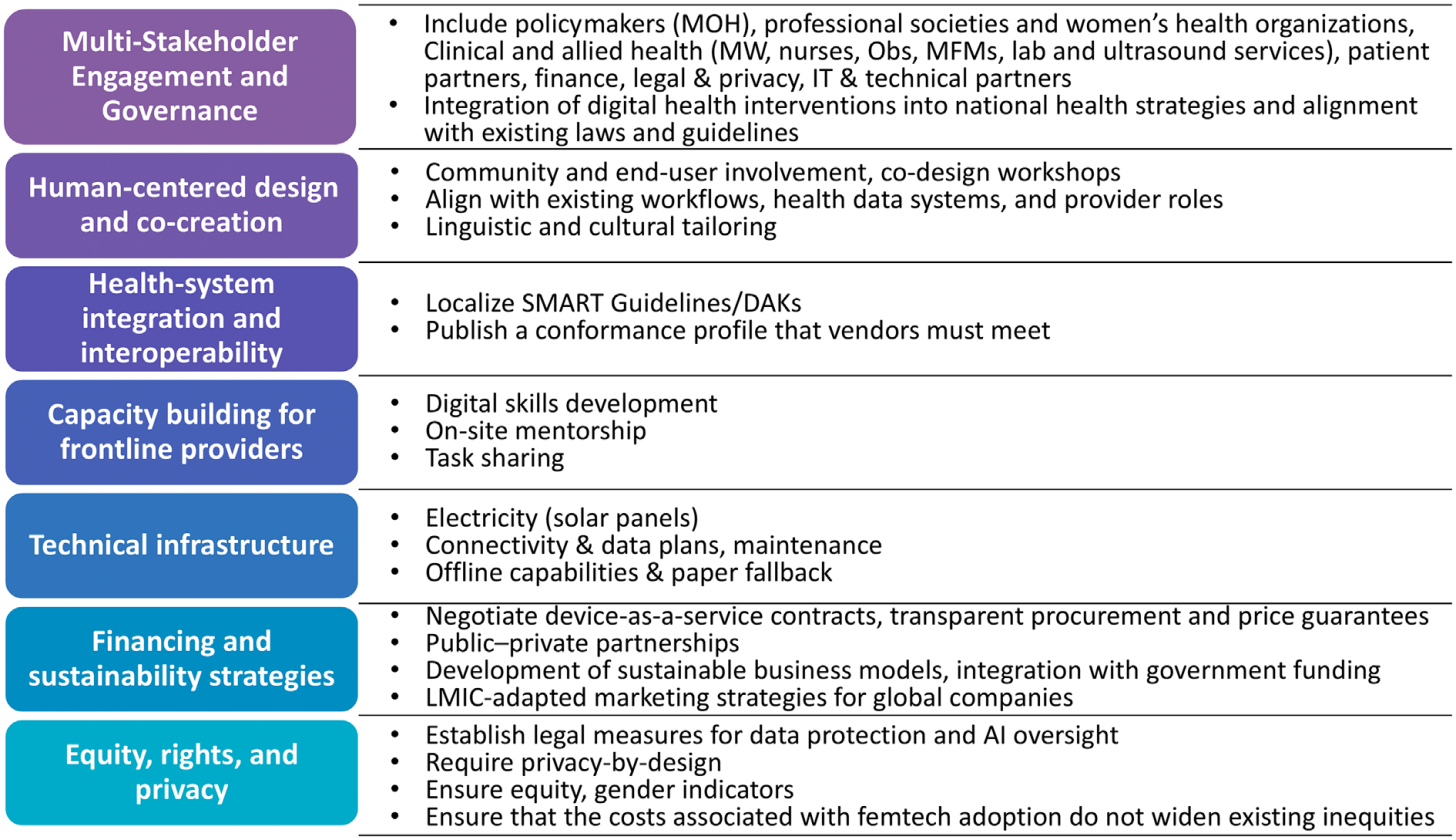
The implementation of FemTech in LMICs should follow several principles and enabling conditions to ensure success and sustainability. These principles also help ensure that FemTech is implemented in a responsible manner that protects patient rights and promotes equity. AI, artificial intelligence; DAK, digital adaptation kit; IT, information technology; LMIC, low‐ and middle‐income country; MOH, ministry of health; MFM, maternal‐fetal medicine; MW, midwives; Obs, obstetricians; SMART, Standards‐based, machine‐readable, adaptive, requirements‐based, and testable.

The implementation of FemTech in LMICs should follow several principles and enabling conditions to ensure success and sustainability. The main principles include multistakeholder engagement and governance, human‐centered design and co‐creation (to increase the chance of consistent adoption of the approach), health‐system integration and interoperability, capacity building for frontline providers, technical infrastructure, financing and sustainability strategies, and equity, rights, and privacy (Figure 4). These principles also help ensure that FemTech is implemented in a responsible manner that protects patient rights and promotes equity.

**FIGURE 5:**
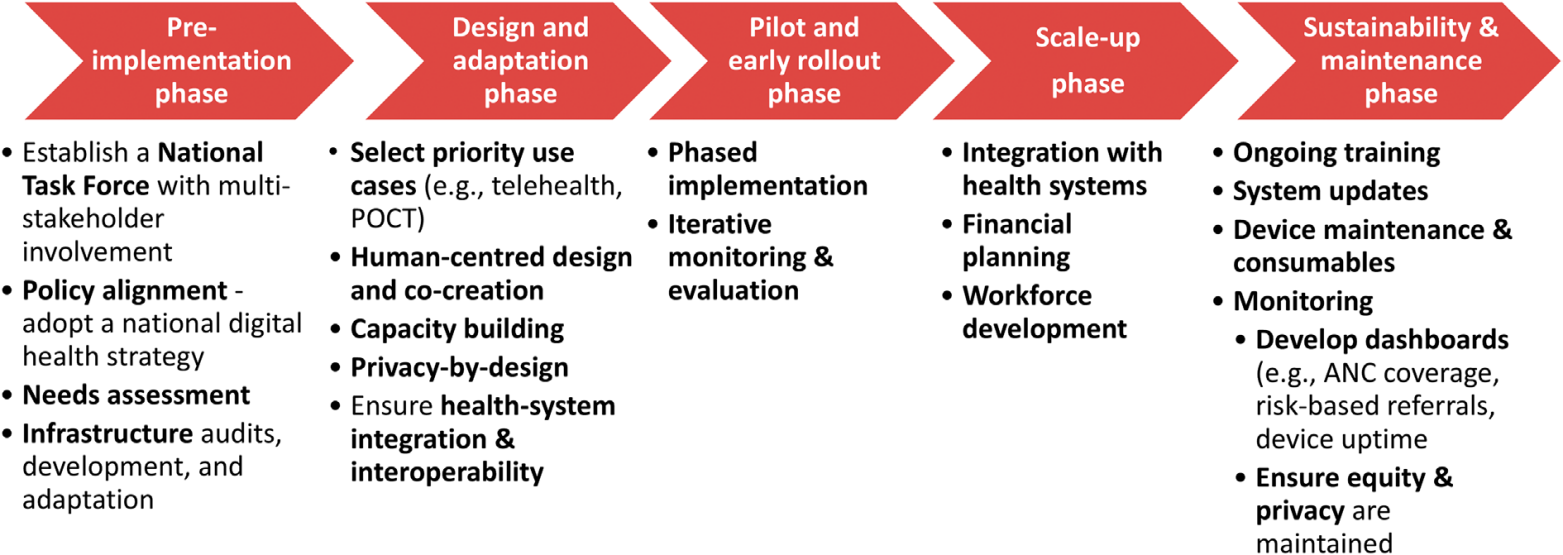
A practical five‐phase framework for the implementation of FemTech in LMICs. This framework is based on the principles and enablers described in Figure 4. These phases are iterative, incorporating feedback loops and monitoring to support adaptation and ensure that benefits reach underserved groups. ANC, antenatal care; LMIC, low‐ and middle‐income country; POCT, point‐of‐care testing.

Finally, we outline a practical five‐phase implementation framework based on these principles and enablers, which includes pre‐implementation, design and adaptation, pilot and early rollout, scale‐up, and sustainability and maintenance (Figure 5). These phases are iterative, incorporating feedback loops and monitoring to support adaptation and ensure that benefits reach underserved groups.

### 1.7 Conclusion

The information reviewed in the current manuscript and the practical framework described above can help countries adopt and adapt selected digital solutions in a safe, equitable, and sustainable manner, with the ultimate goal of improving outcomes for pregnant women and their babies. With thoughtful design that is adapted to local needs and guided by FIGO, FemTech can help expand access to quality care, reduce delays, support providers, and empower women and their families. More evidence is needed to support and guide the implementation of FemTech in LMICs. If implemented effectively and guided by clear principles and a solid framework, FemTech can become a practical tool for safer, more equitable pregnancy care across LMICs.

## 2 TARGET AUDIENCE

This guideline is directed at multiple stakeholders, with the aim of reviewing current evidence on the barriers to the implementation of FemTech in LMICs, and proposing adaptations and strategies aligned with FIGO's guidelines to address these challenges. The target audience comprises the following groups:

- *Healthcare providers*: individuals qualified to care for pregnant women and newborns, including obstetricians, family physicians, midwives, nurses, pediatricians, healthcare educators, and providers operating in remote or geographically isolated outposts.
- *Health system leaders and implementers*: representatives from ministries of health, regional and district health managers, administrators of remote and rural health networks, Indigenous health authorities, health insurance organizations, global health implementing partners, and nongovernmental organizations.
- *Regulators and governance bodies*: national regulatory authorities, data protection and privacy authorities, ethics review committees, and national electronic health (eHealth) or digital health steering committees.
- *Professional and academic organizations*: FIGO and its national member associations, as well as international, regional, and national professional societies relevant to obstetrics and gynecology, ultrasound, family medicine, pediatrics, neonatology, nursing, and midwifery. This group also includes training institutions and researchers in public health, implementation science, and biomedical engineering.
- *Technology and industry partners*: digital health and medical device developers, software and product teams, data scientists, health information system vendors, interoperability and standards specialists, and connectivity providers.

## 3 BACKGROUND

### 3.1 Burden of maternal and neonatal mortality and morbidity in LMICs

Maternal and neonatal mortality and morbidity rates uncover major global health disparities. Despite ongoing efforts, the rates of maternal and neonatal complications remain substantially higher in LMICs compared to HICs. In 2020, WHO reported that the majority (94%) of approximately 300 000 maternal deaths occurred in LMICs, predominantly in South Asia and sub‐Saharan Africa.^1^, ^2^, ^3^ Maternal mortality ratios (MMRs) in LMICs are as high as 400–500 per 100 000 live births, compared with 10–15 per 100 000 live births in HICs.^4^, ^5^ Recent data from 2023 indicate that the lifetime risk of maternal death in LMICs is 1 in 66, in contrast to 1 in 8000 in HICs.^6^ Most maternal deaths in LMICs result from direct obstetric complications, such as postpartum hemorrhage, severe pre‐eclampsia, and sepsis, which are exacerbated by the high rate of predisposing factors, including anemia, non‐communicable diseases (NCDs), and obesity.^7^, ^8^, ^9^, ^10^ These complications also lead to increased rates of both short‐ and long‐term physical and mental health disorders. For most of these deaths, prevention and early recognition are the keys to optimizing the outcomes, and the use of technology can potentially bridge the inequities.

Similar disparities exist in perinatal mortality and morbidity. Of the approximately 2 million annual cases of neonatal death, most occur in LMICs,^11^, ^12^ particularly in sub‐Saharan Africa, where the average neonatal mortality rate reaches 1.7%.^10^ The high neonatal mortality rates are primarily due to preterm birth, intrapartum asphyxia, sepsis, congenital anomalies, and fetal growth restriction. These complications also result in high rates of long‐term complications, such as respiratory disease and neurodevelopmental disability. Stillbirth rates are also disproportionately high in LMICs, with up to 98% of the 1.9 million annual cases of stillbirth worldwide occurring in LMIC settings.^11^, ^13^, ^14^, ^15^

### 3.2 Unmet needs in LMICs

Several factors contribute to the high rates of maternal and perinatal mortality and morbidity in LMICs. Below, we describe some of these unmet needs that lead to inadequate healthcare access and quality, delayed detection, referral, management of pregnancy complications,^16^, ^17^, ^18^, ^19^, ^20^, ^21^ and, ultimately, poor maternal and perinatal outcomes (Table 1).

#### 3.2.1 Incomplete access and coverage

Many women in LMICs receive inadequate pregnancy care, both in terms of frequency and content of antenatal visits, due to a combination of geographic barriers, transportation costs, limited awareness, and sociocultural constraints, such as domestic responsibilities or the need for permission.

#### 3.2.2 Quality and timeliness of care

Even when women do have access to obstetrical care providers, this may not guarantee high‐quality and timely care due to delays in the triage and diagnosis of pregnancy complications, and the lack of critical medications (e.g., magnesium sulfate or anti‐hypertensive drugs), blood products, or essential equipment (e.g., ultrasound or monitoring devices).

#### 3.2.3 Poorer referral and transportation systems

This refers to a breakdown in the structured pathway that ensures a patient at a lower‐level health facility (such as a rural clinic) can be safely and quickly moved to a higher‐level facility (such as a tertiary hospital) when complications arise due to logistical and infrastructure barriers, communication gaps, financial hurdles, or capacity constraints. For example, many areas in LMICs lack ambulance services and have poor roads. Furthermore, out‐of‐pocket transportation costs may be high and unaffordable for many patients. These factors also contribute to the delay in the definitive management of pregnancy complications, often with severe consequences.

#### 3.2.4 Health workforce constraints

Most LMICs experience a shortage of obstetrical care providers (obstetricians and midwives), anesthetists, and neonatologists, further limiting adequate antenatal and postnatal care, as reflected by the large number of unattended home births in LMICs.^11^ Even in settings where staff are available, many providers have insufficient knowledge and experience in managing complicated pregnancies (e.g., severe pre‐eclampsia), obstetric emergencies (e.g., postpartum hemorrhage) due to inadequate training, supervision, and retention.^22^

#### 3.2.5 Infrastructure and logistical limitations

Many facilities in LMICs experience unreliable electricity, which limits their capacity to provide safe surgical and monitoring capabilities, store medications, and implement infection control.^2^, ^12^ In addition, poor water, sanitation, and hygiene (WASH) conditions, inadequate supply chains, and limited access to laboratory or imaging services further limit centers' capacity to provide adequate care.

#### 3.2.6 Data and decision support gaps

The lack of robust health information systems results in unreliable documentation, inadequate early warning systems, and disrupted continuity of care. This also undermines learning and quality improvement efforts.

#### 3.2.7 Sociocultural, religious, educational, and gender norms

Factors such as gender norms, financial dependence, and stigma often limit the ability of women to be involved in health decision‐making, including in the context of pregnancy and the postpartum period. In addition, low levels of education and poor health literacy can make it challenging for women to understand medical recommendations and adhere to complex treatment regimens. Cultural factors and religious practices in LMICs also contribute to high‐risk pregnancies, for example, due to early marriage, adolescent pregnancies, and high parity.^11^, ^22^, ^23^

#### 3.2.8 Financial barriers and sustainability

Many women in LMICs lack insurance coverage, making out‐of‐pocket costs for diagnostics, medications, and transport high and unaffordable. At the health system level, many centers and health organizations lack sustainable financing models, limiting the availability and quality of medical services.

These challenges highlight the urgent need to address core health system deficits and socioeconomic barriers in LMICs, with the ultimate goal of reducing the unacceptable rates of maternal and neonatal morbidity and mortality.

### 3.3 FemTech and advanced technologies as potential solutions for the unmet needs in LMIC

Broadly defined, FemTech, a term coined in 2016 by entrepreneur Ida Tin, refers to a wide range of technology‐based products, mHealth apps, artificial intelligence (AI)‐based tools, and digital platforms and services designed specifically to support women's health.^24^, ^25^ In the context of pregnancy, an increasing number of technologies can support antepartum and postpartum care, even in settings with limited resources. FemTech has the potential to increase access to care through the combination of centralized remote care and self‐monitoring, and enable earlier detection of complications and timely escalation of care. Importantly, many of these interventions can be delivered through mobile technologies that allow pregnant women to interact with digital health systems directly. Using mobile phones, women can report symptoms, receive tailored health guidance, and participate in remote monitoring programs in near real time. These changes can, in turn, improve pregnancy outcomes, reduce the number of in‐person antenatal care visits (thereby increasing patient compliance and convenience and reducing patient and healthcare system costs), overcome socioeconomic barriers, and promote equity in access to and quality of care.

The global investment and uptake of FemTech accelerated during COVID‐19.^26^ These were supported by initiatives such as WHO's guideline on digital interventions for health system strengthening and the SMART (Standards‐based, Machine‐readable, Adaptive, Requirements‐based, and Testable) guidelines, which provide a framework for the standardized adoption of digital health tools in antenatal care.^27^, ^28^

The benefits of FemTech are expected to be greatest in LMIC settings, given the standard care challenges and other unmet needs described above, together with a high rate of pregnancy complications (Table 1, Figure 1). However, the uptake of FemTech in LMICs remains limited, primarily due to issues such as affordability, power, connectivity, regulation, digital literacy, and social and cultural factors. Importantly, many international guidelines on antenatal care and pregnancy complications, including those advocating the implementation of advanced technologies, often omit LMIC‐specific considerations and fail to provide context‐specific strategies and adaptations suitable for constrained health systems.^29^, ^30^, ^31^ This gap underscores the need to develop strategies for the implementation of FemTech in LMIC settings. FIGO and its partners are well positioned to coordinate the development of such dedicated global guidance tailored to resource‐limited settings (Box 1).

BOX 1 **What is FemTech?**

**Table:** 

Technology‐based products, mobile apps, artificial intelligence tools, and digital platforms specifically designed to support women's health Goals in pregnancy: Increase access to care Combine centralized remote care with self‐monitoring Detect complications earlier Escalate care quickly when needed

### 3.4 Purpose of this document

The aim of this document was to review the current evidence on the benefits of FemTech for improving antenatal care and pregnancy outcomes, describe the challenges involved in the implementation of FemTech in LMICs, and propose FIGO‐aligned adaptations and strategies to resolve these challenges and promote equity, along with a practical framework for the implementation of these strategies. This document should be understood as a strategic guidance paper, developed under the direction of the FIGO Committee on Women’s Health and Technology, rather than a formal systematic review or clinical practice guideline. It is based on available evidence and the consensus of an international group of experts to provide FIGO‐aligned, practical recommendations for stakeholders in resource‐limited settings.

## 4 FemTech FOR PREGNANCY CARE: EVIDENCE OF BENEFITS

Pregnancy‐related FemTech solutions can be classified into five broad, complementary categories: (1) mobile apps and SMS‐based communication tools for patient education, health tracking, reminder, and communication; (2) wearables and home‐use devices and sensors, for monitoring of BP, heart rate, glucose, maternal weight, fetal heart rate, and uterine contractions; (3) POCT devices, such as handheld ultrasound, and tests for conditions such as anemia, HIV, syphilis, preterm birth risk, and pre‐eclampsia, which can be used at home or in local facilities; (4) AI‐powered tools for education and training, decision support, risk stratification, improved dynamic prediction and diagnosis, and automated image analysis; and (5) digital platforms, such as telemedicine and remote counseling, which support centralized antenatal care models with trained providers and AI‐based decision systems.

Together, these technologies can improve access to antenatal care, improve care quality, bring diagnostics closer to patients, standardize decision‐making, and streamline the escalation of care when needed. Still, it is important to be mindful of the quality and level of scientific rigor behind specific technologies and digital tools. In particular, a distinction should be made between technological capability (what a device can theoretically do) and proven clinical effectiveness (actual improvements in maternal or perinatal outcomes), as the strength of evidence differs substantially across FemTech categories (Box 2). In this section, we summarize available evidence on the benefits of selected FemTech tools and products (Table 2) and describe how these tools can be used in antenatal care for both low‐ and high‐risk pregnancies (Figures 2 and 3).

BOX 2 **The five main categories of pregnancy FemTech.**

**Table:** 

Mobile apps and SMS tools for patient education and health tracking Wearables and home devices to monitor blood pressure, glucose, and heart rate Point‐of‐care testing devices like handheld ultrasounds and rapid infection tests Artificial intelligence tools for decision support and risk scoring Digital platforms for telemedicine and remote counseling

### 4.1 Mobile apps and SMS‐based communication

Mobile apps and SMS‐based tools are among the most widely studied FemTech applications, with strong evidence supporting their effectiveness. These digital tools can deliver timely educational content in local languages, facilitate symptom tracking with alerts for danger signs, provide appointment reminders and support actions to be taken, and enable two‐way messaging with healthcare providers. WHO's digital interventions guideline and SMART guidelines specify key design requirements for these digital tools in the context of antenatal care to ensure standardized, consistent implementation of these apps and platforms across countries.

Beyond reminders and educational messaging, mobile platforms can also enable real‐time symptom reporting and dynamic risk communication. Pregnant women can report warning signs such as reduced fetal movements, headache, bleeding, or swelling through structured mobile interfaces, triggering automated triage guidance or alerts to healthcare providers. Such bidirectional communication models may help shorten delays in care seeking and strengthen early detection of pregnancy complications.

Several studies have reported significant improvements in antenatal care utilization, skilled birth attendance, and patient satisfaction and engagement with these interventions.^32^, ^33^, ^34^, ^35^, ^36^, ^37^ It should be noted, however, that much of this evidence pertains to process and utilization outcomes rather than hard clinical endpoints such as maternal or perinatal mortality. One example is South Africa's MomConnect platform, which illustrates the feasibility of implementing interactive mobile messaging and SMS‐based educational programs at the population level.^38^, ^39^ These tools have been shown to be especially useful in women with gestational diabetes, where the use of mobile apps for the self‐tracking of glucose and lifestyle, along with provider feedback, was associated with improved glycemic control, reduced insulin requirements, decreased gestational weight gain, and lower rates of macrosomia.^33^, ^40^, ^41^, ^42^ Recent systematic reviews and meta‐analyses suggest that such digital tools can improve healthcare delivery, including increased antenatal care visits, adherence to immunization schedules, facility‐based deliveries, and breastfeeding,^43^, ^44^, ^45^ as well as patient‐reported outcomes.^45^, ^46^, ^47^

### 4.2 Wearables and home‐use devices

Wearable technologies and home‐use devices can improve self‐monitoring, support remote centralized care, and potentially improve clinical outcomes, especially in high‐risk pregnancies.

Home BP cuffs with telemonitoring have been widely studied. In two large randomized trials (BUMP 1 and 2) involving high‐risk pregnancies in the UK, home BP monitoring with telemonitoring was found to be safe, although it did not significantly accelerate the time to a formal clinic‐based diagnosis of hypertension by healthcare providers compared with standard care.^48^, ^49^ These studies highlight the importance of pairing home monitoring with effective and timely care pathways. Economic analyses of home BP telemonitoring indicated that the cost implications of these interventions depend on workflow design, specifically whether the remote monitoring is used to safely replace routine in‐person clinic visits or is simply added as an extra expense on top of standard care.^50^, ^51^

For diabetes in pregnancy, the use of Bluetooth‐enabled glucometers has been associated with lower rates of pre‐eclampsia and neonatal hypoglycemia.^52^ Advanced technologies, such as continuous glucose monitoring (CGM) and hybrid closed‐loop systems, have been associated with improved glycemic control and lower rates of neonatal complications.^53^ In case of high‐risk pregnancies, such as in patients with type 1 diabetes, the use of CGM improved maternal glycemic control and reduced neonatal complications, including large‐for‐gestational‐age and neonatal intensive care admissions.^54^, ^55^ For women with gestational diabetes, there is growing evidence that CGM can improve glycemic metrics, gestational weight gain, and infant birth weights.^56^

Monitoring of maternal gestational weight gain through smart weight scale devices has been shown to improve gestational weight management. Studies that tested interventions incorporating Bluetooth‐enabled home scales reported significant reductions in postpartum weight gain.^57^, ^58^, ^59^

The diagnostic accuracy of home fetal monitoring technologies, including wireless cardiotocography and home ultrasound devices, has been reported to be comparable to that of standard clinical devices.^60^, ^61^ For example, mobile cardiotocography achieved a high agreement with standard CTG for antepartum monitoring (91%, kappa 0.60).^62^ Simple, beltless fetal heart rate monitors can be used at home to assess fetal well‐being in high‐risk pregnancies. For example, the Monica Novii/Novii+ wireless patch fetal heart rate monitor has regulatory approval and supporting validation studies.^61^ Similarly, home ultrasound demonstrates high user satisfaction and detection rates for fetal viability, fetal movements, and amniotic fluid volume.^63^

Emerging wearable tocometers (including devices using electrophysiological approaches) that are coupled with advanced algorithmic detection systems are available, but additional evaluation and validation are still needed.^64^, ^65^, ^66^, ^67^

These devices can also be used to adjust medications or trigger remote consultation or urgent referral. They have a role in the routine antenatal care of low‐risk pregnancies, but are especially relevant for conditions such as hypertensive disorders, diabetes, preterm birth, and fetal growth restriction. In addition, these devices offer the potential for individual‐level experimentation (n‐of‐1 studies), a research design that focuses on serial measurements from a single individual over time. The rationale behind this methodology is that an individual's threshold for disease or responses to interventions can vary significantly, and aggregate data from larger studies may not capture meaningful individual‐level changes. Therefore, wearables and home‐use devices lay the foundation for personalized diagnostic and therapeutic interventions in pregnancy.^68^, ^69^, ^70^, ^71^

### 4.3 Point‐of‐care testing (POCT) devices

POCT technologies can enable task‐shifting, improve diagnostic accuracy, and facilitate timely diagnosis and management of pregnancy complications, especially in low‐resource settings.

WHO recommends the use of rapid dual HIV/syphilis diagnostic tests as the first‐line option in prenatal care because they are simple, cost‐effective, and enable same‐day diagnosis and treatment.^72^ Many countries have adopted this policy, and recent assessments have confirmed improved coverage and cost savings.^73^, ^74^, ^75^ A recent study reported that syphilis POCT resulted in a dramatic 93% reduction in the rate of congenital syphilis.^75^

POCT anemia testing with devices like HemoCue supports screening in peripheral settings and is currently widely used. The accuracy of these devices has been reported to vary depending on the device, sample type (capillary vs. venous), and environmental conditions.^76^, ^77^, ^78^

In women with suspected threatened preterm labor, vaginal or cervical fetal fibronectin has been shown to have a high negative predictive value for preterm birth, which helps avoid unnecessary admissions and transfers of patients with a negative result.^79^, ^80^, ^81^ Similarly, in patients with suspected pre‐eclampsia, POCT using assays such as soluble fms‐like tyrosine kinase‐1 (sFlt‐1) and placental growth factor (PlGF) improves risk stratification and time to diagnosis, reduces unnecessary admissions, referrals, and interventions, and facilitates timely diagnosis of pre‐eclampsia. Several validated point‐of‐care platforms are available, including newer whole‐blood assays that require as little as 20 μL obtained from a finger prick, thereby eliminating the need for centrifugation or venipuncture.^82^, ^83^, ^84^, ^85^

Handheld ultrasound has advanced rapidly. Recent data suggest that portable handheld ultrasound devices can obtain obstetric biometry and estimate gestational age with clinically acceptable accuracy.^86^, ^87^, ^88^ A recent study in which midwives were trained to perform basic obstetric point‐of‐care ultrasound (POCUS) in rural areas using a tablet platform and mobile phone transmission reported high accuracy compared with standard methods and decreased turnaround times.^62^ When paired with AI (see below), these low‐cost, battery‐powered devices can increase access to dating and growth assessments in facilities that lack expert sonographers.

### 4.4 Artificial intelligence (AI)‐powered tools

AI‐powered platforms and devices are another rapidly evolving category.^89^ AI‐based tools can support patient and provider education. AI‐assisted conversational tools and adaptive learning platforms may expand access to credible medical content. Similarly, AI‐powered educational models and simulations can improve provider knowledge and skills. In addition, AI‐enabled conversational interfaces using natural language processing may provide accessible, culturally adapted guidance to pregnant women, particularly in settings with limited health literacy. These tools can deliver context‐specific health information, answer frequently asked questions, and guide users through symptom assessment pathways while directing them to appropriate care when warning signs are identified. However, implementation of such tools requires oversight and adherence to WHO guidance on AI ethics and safety.^90^

AI models that are based on routinely collected serial data from apps, wearables, and home devices (e.g., symptoms, vital signs, fetal heart rate) can enable dynamic prediction and diagnosis of pregnancy complications such as pre‐eclampsia and fetal complications.

AI‐powered decision‐support tools integrated into electronic medical records can improve adherence to guidelines and the quality of antenatal care.^91^ Examples in LMIC settings include Tanzania's Nurse Assistant and the Pregnancy and Newborn Diagnostic Assessment (PANDA) apps.^92^, ^93^ These platforms guide frontline health workers through standardized antenatal visits by providing step‐by‐step clinical protocols and automated risk alerts. Consequently, they have been shown to improve provider adherence to guidelines, reduce documentation burden, and increase the overall quality and comprehensiveness of antenatal care. Recent systematic reviews of AI‐augmented clinical decision support systems in pregnancy highlight the potential of these tools to enable dynamic prediction of complications and improve adherence to clinical guidelines, but emphasize the need for rigorous evaluation of their impact and equity.^91^, ^94^, ^95^

In imaging, AI can automate the interpretation of limited or “blind sweep” ultrasound, guiding non‐expert users to obtain images that can be used to estimate gestational age, obtain basic biometry, and detect fetal structural anomalies.^87^, ^88^, ^96^, ^97^, ^98^, ^99^, ^100^, ^101^ In a recent study conducted in Zambia and North Carolina, the research team used handheld probes (Butterfly iQ) with an AI tool integrated in a low‐cost, battery‐powered device. The device was capable of estimating gestational age from blind ultrasound sweeps with accuracy comparable to that of trained sonographers.^88^ The development of tele‐ultrasound capabilities, along with the automation of other measurements (cervical length and fetal anomaly detection), will further increase the potential impact of AI‐enabled POCUS. By enabling minimally trained community health workers to perform complex anatomical and risk screenings that typically require scarce specialists, these tools can bring HIC‐level diagnostic accuracy directly to underserved LMIC communities.

### 4.5 Digital platforms: Telemedicine, remote counseling, and centralized antenatal care models

Digital platforms and telemedicine interventions can reduce healthcare resource utilization and costs while maintaining clinical safety. Telemedicine can facilitate hybrid care, where routine contacts, data review, and counseling occur virtually, while in‐person visits are focused on physical examinations, imaging, and labs. When combined with wearables, home devices, and AI‐supported decision systems, these platforms can further enable centralization of care, thereby promoting equitable access and quality of care, especially in remote and rural areas. Other benefits include patient convenience and reduced costs for patients and the healthcare system. In a recent FIGO publication, Hod et al. described a framework for an innovative hybrid e‐health model of antenatal care for low‐risk pregnancies (PregCare) that can help transform the traditional model's high frequency of office visits into a safer, reduced‐visit approach. This model is based on increasing virtual connections, the use of advanced technologies for point‐of‐care and self‐care, and centralized support from doctors, nurses, and community‐based providers.^102^

The randomized OB Nest trial showed that a hybrid telemedicine‐based antenatal care model reduced clinic visits, increased patient satisfaction, and reduced prenatal stress without compromising clinical outcomes.^103^ Recent reviews concluded that telehealth in antenatal care can maintain care quality and access, with no signal of harm for common outcomes.^104^ In LMIC settings, digital pregnancy care platforms with integrated decision support have improved the content and quality of antenatal care, reduced documentation burden, and enabled quality‐improvement dashboards.^91^, ^105^, ^106^

### 4.6 Application of FemTech in low‐ and high‐risk pregnancies

Evidence across all the FemTech categories described supports their integration into maternal and perinatal care. The evidence is particularly strong for mobile apps/SMS interventions and digital platforms/telemedicine, somewhat weaker for wearables and POCT devices, and is emerging for AI‐powered tools. The most compelling benefits are likely to be achieved when these technologies are combined to provide a holistic, multidisciplinary antenatal care approach for low‐ and high‐risk pregnancies. For example, SMS/app education to increase attendance, home BP or continuous glucose monitoring devices combined with a telemedicine platform to optimize management, POCT to confirm diagnoses at the local facility, and AI decision support to standardize triage and referral (Figures 2 and 3). WHO's 2019 digital guideline underscores that the effectiveness of these tools depends on how they are implemented.^27^

In low‐risk pregnancies, the main role and potential benefits of these tools are to increase access to routine antenatal care, reduce the number of in‐person visits and the associated logistical and financial burden on families and health systems, facilitate risk stratification, and ensure timely detection of complications (Figure 2). Mobile apps and SMS reminders can be used to improve adherence to recommended antenatal schedules, enhance maternal knowledge, and encourage healthy behaviors such as adequate nutrition and recognition of danger signs. Wearables and simple home‐use devices, such as digital BP monitors or weight scales, can allow women to self‐monitor their health with remote oversight by their care providers. Hybrid antenatal care models have been shown to reduce travel costs and improve patient satisfaction, particularly in rural or underserved areas. These strategies free up limited health resources, reduce the burden on health facilities, and ensure equitable access to essential care for low‐risk populations. A recent paper from FIGO provides a framework for a new hybrid model that can reduce the number of in‐person visits in low‐risk pregnancies from 14 (according to traditional models^107^) to only 6–8 visits.^102^

For women with high‐risk pregnancies, such as those with hypertensive disorders, diabetes, past pregnancy complications (e.g. past preterm birth), multiple pregnancies, or fetal growth restriction, the potential value of FemTech is even greater (Figure 3). Continuous or frequent monitoring with wearables (such as home BP monitors, continuous glucose monitors, or fetal heart rate sensors) can enable early detection of deterioration. AI‐powered risk‐stratification tools can process continuous data streamed from home devices to trigger alerts and guide escalation to higher levels of care. Point‐of‐care diagnostics, such as portable ultrasound or rapid tests for pre‐eclampsia and preterm birth, can be used at local centers or even at home to avoid unnecessary transfers while ensuring timely diagnosis and referral. Telemedicine and centralized care platforms that connect frontline providers with specialists can facilitate timely support when managing complex cases, thereby increasing access to high‐quality care. The integration of these technologies into the management of high‐risk pregnancies has the potential to improve maternal and perinatal outcomes substantially.

## 5 CHALLENGES, BARRIERS, AND PROPOSED ADAPTATIONS TO IMPLEMENTING FemTech IN LMICs

FemTech has the potential to reduce the disparity in antenatal care and pregnancy outcomes. In practice, implementing these digital platforms and technologies in LMIC requires careful consideration of many barriers related to infrastructure, technology, economics, sociocultural factors, and regulations. Below, we outline the main challenges found in digital health and maternal health research (Box 3). For each challenge, we propose adaptations and strategies to address them (Table 3).

BOX 3 **Major barriers and key adaptations for implementing FemTech in low‐ and middle‐income countries.**

**Table:** 

*Infrastructure and connectivity*: Barriers like intermittent electricity and poor Internet can be addressed using solar or battery backups, hybrid connectivity with offline capabilities, and community internet hubs *Digital literacy and workload*: Challenges, including low digital literacy and overburdened staff, can be mitigated through training programs, task‐shifting, and creating simple, user‐friendly interfaces that fit seamlessly into existing workflows *Policy and regulation*: Concerns regarding data privacy and weak legal frameworks require comprehensive policy advocacy, privacy‐by‐design approaches, data encryption, and clear ethical guidelines for AI *Sociocultural factors and equity*: To overcome gender norms, language barriers, and lack of device access, solutions should involve community co‐creation, culturally sensitive designs, device subsidies, and strict equity monitoring *Cost and sustainability*: The high costs of technology and reliance on short‐term pilot funding can be resolved through innovative financing models like bulk purchasing, local manufacturing, and public‐private partnerships

### 5.1 Infrastructure, connectivity constraints, and device maintenance

Infrastructure limitations are among the main barriers to the implementation of FemTech in low‐resource settings. Many areas in LMICs experience intermittent or no electricity, weak mobile and Internet connectivity (especially in rural and remote areas), and limited access to devices such as smartphones, tablets, and sensors.^108^, ^109^ These deficits necessitate digital solutions with offline capabilities and low bandwidth requirements.^110^, ^111^, ^112^, ^113^

Even when devices and sensors are available, their maintenance remains a major challenge due to frequent calibration requirements, shortages of spare parts and consumables driven by cost and unreliable supply chains, short battery life, and limited accuracy and durability due to environmental factors such as heat, humidity, and dust.^111^, ^114^

Another barrier related to infrastructure is the poor interoperability of health information systems. FemTech tools often need to link with existing health information systems, such as electronic medical records. In many LMICs, these systems are often non‐existent, fragmented, and non‐standardized, which complicates data exchange and limits the integration of FemTech solutions.

Several practical strategies have been suggested to address these infrastructure challenges. Solar power solutions are effective in many settings, with studies showing that solar‐powered chargers and lights can support digital diagnostics in remote areas.^110^ Solar panels have also been shown to enable ultrasound equipment operation in rural locations.^111^ Mobile tools should support hybrid connectivity (offline caching, SMS fallback) and have solar or battery backup, while health systems should have adequate contingencies, such as device caching and paper backup. Real‐world initiatives such as UNICEF's Oky app and the mMitra program perfectly illustrate how these tailored, hybrid digital innovations can successfully operate in the low‐connectivity and low‐resource conditions common to many LMIC settings.^115^, ^116^ Devices should be adapted to local settings to ensure voltage compatibility and functionality in low‐resource environments.^112^, ^117^ Community Internet hubs and shared digital spaces can improve access in remote areas. Furthermore, the rapid expansion of Low Earth Orbit (LEO) satellite internet constellations, such as Starlink, holds transformative potential to bridge the global digital divide. By providing high‐speed, low‐latency broadband to geographically isolated regions without requiring extensive ground infrastructure, these satellite networks can reliably connect remote clinics to centralized health systems. Device maintenance can be facilitated by using durable, field‐tolerant hardware, planning for spare parts, and building local repair networks. To improve interoperability, digital solutions should follow international data standards and modular architectures (e.g., OpenHIE).^118^, ^119^

### 5.2 Digital literacy, provider workload, and human acceptance

The potential users of FemTech products in pregnancy, including pregnant women, community health workers, midwives, and clinicians, may find it challenging to use digital tools due to low literacy, limited experience with smartphones or apps, language barriers, and poorly adapted interfaces. These challenges, along with cultural resistance or distrust toward new technologies, may reduce uptake and engagement.^113^, ^120^, ^121^, ^122^, ^123^, ^124^, ^125^, ^126^, ^127^, ^128^ Another factor that may limit uptake of new digital solutions is that many health workers already operate at full capacity.^129^ The introduction of new systems can increase their workload (e.g., data entry, troubleshooting, alerts) and disrupt their workflows.^110^, ^125^, ^126^, ^127^, ^130^

Several strategies have been proposed to address these challenges, including local capacity building, training programs, and mentorship. User‐friendly manuals and interfaces are crucial, and both women and care providers should be engaged in designing these tools from the start.^110^, ^111^, ^120^, ^121^, ^128^, ^131^, ^132^, ^133^ Particularly in settings where traditional text literacy is low or language barriers exist, the use of image‐based technologies may be valuable. Implementing adaptive interfaces that utilize locally relevant icons, visual cues, and voice prompts rather than text can significantly bypass literacy constraints and improve equitable access. Studies from India show that strong physician leadership was key to the successful implementation of fetal heart rate monitoring.^121^, ^131^ For better adoption, digital tools should be simple, minimize extra steps, fit well into existing workflows, automate data capture, and provide real benefits to users, such as time savings or decision support. The use of incentives, such as financial rewards, recognition, or performance measures, can also improve uptake. Task‐shifting, such as delegating responsibilities to less specialized health workers, has been shown to be effective in addressing staff shortages.^121^, ^134^ Ultimately, fostering long‐term acceptance requires integrating digital health and FemTech competencies into the core curricula of medical and midwifery schools, ensuring that the next generation of healthcare providers is trained to utilize these tools as a standard component of maternal care from the earliest stages of their education.

### 5.3 Policy, regulation, and legal and ethical barriers

Maternal health data are sensitive, and concerns about patient privacy, consent, and how the data might be used are common when new digital tools are introduced. These concerns are even greater in settings without strong regulatory frameworks and data protection laws.^114^, ^117^, ^135^ The lack of supporting policy and clear regulatory processes might reduce user trust and create legal and ethical barriers to the implementation of FemTech.^110^, ^125^, ^127^

Addressing these regulatory issues requires a comprehensive approach that involves strong policy advocacy and the development of a legal framework that provides clear guidance for FemTech implementation and patient data protection.^113^ Policy advocacy is essential to raise awareness among policymakers about the potential benefits and challenges of FemTech implementation. Data privacy should be protected even in settings with limited resources or Internet access through privacy‐by‐design and encryption. The ethical use of AI and digital health tools requires clear rules for consent, the collection of only necessary data, the protection of information, and adherence to WHO and FIGO guidance on these issues.^90^, ^109^, ^135^, ^136^

### 5.4 Sociocultural, religious factors, and equity

Even within LMICs, there are substantial gaps in access to digital tools. Women in remote, lower‐income, or marginalized communities may not have smartphones, data plans, or the skills to use digital technology. Cultural attitudes and gender norms can also limit women's ability to access or use technology independently.^120^, ^137^, ^138^, ^139^ In LMICs, women are approximately 15% less likely than men to use mobile Internet, which means that more than 800 million women have limited access to online digital health tools.^140^, ^141^ Language barriers and low health literacy make it even harder for some women to use these tools, especially in multilingual or low‐literacy settings.^120^, ^122^, ^123^, ^124^, ^127^, ^128^ Religious beliefs and the influence of preachers can also affect the behaviors of pregnant women and their uptake of some of these technologies.

If digital solutions are not designed with equity in mind, they may end up widening disparities rather than narrowing them. The design and implementation of FemTech interventions should involve communities and include adaptations to local cultures to overcome social barriers. Possible strategies include awareness campaigns, working with local leaders, and using local knowledge, culturally sensitive design principles, and social media to change attitudes and norms.^112^, ^132^, ^134^, ^138^ Programs should prioritize women in rural areas and marginalized groups, paying attention to issues such as access to devices, data plan costs, and how social norms may affect women's access to and use of phones. Specific approaches that can be used to address the gender gap include device subsidies, establishing community access points, adding safety features, and monitoring uptake by sex, age, location, and income. International groups like GSMA are working to make devices more affordable and expand mobile Internet use.^141^

### 5.5 Cost, affordability, and sustainability

Cost and affordability are another major barrier to the implementation of FemTech in LMICs. Many pregnancy digital health initiatives begin as pilot programs but fail to transition to sustainable, large‐scale solutions. Main challenges include the high costs of technology or device acquisition, consumables (e.g., CGM sensors and patches), user fees, and maintenance.^111^, ^112^, ^134^ These costs are even harder to manage in the absence of clear models for revenue or reimbursement, limited support from local institutions, loss of interest from donors, and insufficient alignment with national health plans.^117^, ^125^, ^126^, ^131^ In addition, many women in these countries have limited insurance coverage and thus face considerable out‐of‐pocket expenses, further limiting access to these technologies and services.

These financial challenges emphasize the importance of sustainable funding, stakeholder involvement, and alignment with health policy plans. Innovative financing models have shown success in overcoming some of these economic barriers.^142^ Examples include bulk purchasing, subsidized pricing models, partnerships between public and private groups, and local manufacturing initiatives.^113^, ^117^, ^125^, ^130^, ^134^

## 6 GETTING IT RIGHT: A PRACTICAL FRAMEWORK FOR IMPLEMENTING FemTech IN LMICs

Closing the gap in maternal and perinatal outcomes between LMICs and HICs cannot be achieved by technology alone. For FemTech to be successfully implemented in LMICs, a comprehensive, practical framework that involves all relevant stakeholders is needed.^130^, ^143^, ^144^, ^145^, ^146^, ^147^, ^148^, ^149^, ^150^ This getting‐it‐right approach should: (1) align digital tools with national priorities; (2) translate best‐practice care into executable tools based on FIGO guidelines and best‐practice advice on the management of pregnancy complications (diabetes, hypertension, obesity, fetal growth restriction, preterm labor, and postpartum care) across the antenatal, intrapartum, and postpartum periods;^136^, ^151^, ^152^, ^153^, ^154^, ^155^, ^156^, ^157^, ^158^, ^159^ (3) invest in people and infrastructure; and (4) protect patients' rights. Implementation should follow recent WHO guidance, which outlines a clear path from policy to practice, including national digital health plans, implementation toolkits, and software‐neutral clinical content that countries can adapt as needed.^27^, ^28^, ^90^, ^160^, ^161^

This section provides practical guidance in two parts. First, we describe the guiding principles and enabling conditions needed for the successful implementation of FemTech (Figure 4). Second, we convert these principles into a five‐step implementation pathway, from preparation to long‐term sustainability, while monitoring impact to ensure that benefits reach underserved groups without widening existing inequities (Figure 5).

### 6.1 Guiding principles and enablers

The implementation of FemTech in LMICs should follow several principles to ensure success and sustainability (Box 4). These principles also help ensure that FemTech is implemented in a responsible manner that protects patient rights and promotes equity (Figure 4).

BOX 4 **Principles for equitable FemTech design in pregnancy.**

**Table:** 

Build a National Task Force that includes government, health workers, patients, and tech experts Use simple, user‐centered interfaces suited to low literacy Systems should be low‐cost, low‐bandwidth, and offline‐enabled Integrate with existing health systems and national priorities Protect data privacy and ethical standards Prioritize equitable access for underserved women

#### 6.1.1 Multi‐stakeholder engagement and governance

Successful FemTech programs require engagement of multiple stakeholders at the policy, organizational, and community levels.^130^, ^148^, ^162^, ^163^, ^164^, ^165^, ^166^ This can be achieved by creating a National Task Force that brings together: (1) representatives from the Ministry of Health, national digital health units, and other policymakers; (2) professional societies and local women's health non‐governmental organizations, and advocacy groups; (3) a wide range of healthcare providers, including midwives, nurses, family doctors, obstetricians, maternal‐fetal medicine specialists, representatives of laboratory and radiology services, public health workers, and referral hospitals; (4) patient partners, including pregnant and adolescent women; (5) health educators in medical and midwifery schools; (6) leaders in finance, health economics, and procurement; (7) legal and privacy experts, such as data protection officials and ethics board members; and (8) information technology and technical partners, including software vendors and mobile network operators.

#### 6.1.2 Human‐centered design and co‐creation

Digital tools and technologies should be easy to use, useful, and trusted by those who use them. Involving communities and users is key to successful implementation.^167^, ^168^ Co‐design with women, adolescents, midwives, nurses, community health workers, and obstetricians can help adjust language, support different literacy levels, and add safety features to ensure these interventions fit with cultural values and meet real user needs.^162^, ^169^ These tools should also be aligned with existing health system workflows and staff roles to avoid work overload and ensure these technologies meet real‐life constraints such as intermittent power, connectivity, and shared devices.^146^, ^170^

#### 6.1.3 Health‐system integration and interoperability

New digital tools should fit national antenatal and postnatal care health systems. Countries can adapt the WHO SMART guidelines and their Digital Adaptation Kits for antenatal care to create shared, software‐neutral content for digital platforms.^28^, ^171^, ^172^ Countries should also publish national requirements that vendors must follow. To achieve true seamlessness, stakeholders should push for the adoption of common healthcare IT platforms and universal data standards, similar to the DICOM standard for medical imaging. The use of open architectures and international standards, such as HL7 FHIR, can facilitate data exchange across centers and the effective integration of home devices, POCT, and imaging into shared records across all levels of care.^118^, ^119^

#### 6.1.4 Capacity building for frontline providers

Digital literacy and training play an important role in whether new digital solutions are successfully adopted over time. Ongoing training in digital skills, local hands‐on mentorship, and clear roles for sharing tasks can help providers adopt these tools with confidence.^173^

#### 6.1.5 Technical infrastructure

The availability of reliable power, Internet connectivity, and device maintenance is essential for safe and effective use of digital solutions. In settings with intermittent electricity and network access, digital tools and health systems should support offline capabilities with synchronization and a paper‐based backup. Funding plans should include provisions for sustained electricity (e.g., solar power and batteries), connectivity (through community WiFi and data plans), consumables, and local maintenance support.^174^, ^175^

#### 6.1.6 Financing and sustainability strategies

Sustainable digital solutions require ongoing funding that covers all costs associated with large‐scale implementation, not just pilot projects. Governments and partners should develop sustainable business models, promote public‐private partnerships, adopt transparent bulk procurement, and establish device‐as‐a‐service contracts.^176^ Obtaining support from professional organizations and local leaders can also help sustain these programs over time.

#### 6.1.7 Equity, rights, and privacy

Legal measures are needed to protect women from data misuse and stigma. Patient data should be protected through a privacy‐by‐design approach, informed consent, and clear oversight of any AI‐enabled features.^90^, ^136^ Tools should be adapted for low literacy and affordability, and programs should monitor uptake within specific groups (e.g., rural and marginalized groups) and track gender equity indicators to ensure that FemTech adoption narrows rather than widens existing inequities.

### 6.2 Implementation framework: A five‐phase approach

Evidence shows that effective FemTech implementation is most successful when it follows a structured, phased approach that is adapted to local needs and involves all key stakeholders. Below, we outline a practical five‐phase implementation framework based on the principles and enablers described in the previous section, progressing from preparation to long‐term sustainability (Figure 5). These phases are iterative, incorporating feedback loops and monitoring to support adaptation and ensure that benefits reach underserved groups (Box 5).

BOX 5 **Practical steps for implementing FemTech in low‐ and middle‐income countries.**

**Table:** 

Assess local needs and readiness Co‐design solutions with communities and providers Pilot and evaluate feasibility Integrate with national health systems Scale sustainably while monitoring equity

#### 6.2.1 Phase 1: Pre‐implementation

The first step is to establish a National Task Force with broad multistakeholder representation. The task force should conduct needs assessments and infrastructure audits to identify priority use cases, technical gaps, and policy requirements. It should also ensure that FemTech priorities are aligned with national digital health strategies and antenatal care programs. This preparatory phase should yield a clear, well‐funded roadmap for FemTech implementation.

#### 6.2.2 Phase 2: Design and adaptation

The next step is to select several high‐priority use cases (e.g., telemedicine, POCT, or digital registries) and design corresponding interventions. Co‐design workshops should involve end users to adapt content to local context, languages, and workflows. Patient privacy and interoperability should be incorporated from the initial design stage. This phase should also focus on capacity building to ensure that teams are equipped to implement and use the new interventions effectively.

#### 6.2.3 Phase 3: Pilot and early rollout

The initial implementation should be flexible and follow an iterative process that involves user feedback and monitoring, with adjustments to the interventions made as needed. Lessons from this pilot phase can help guide decisions about scaling up and inform adaptations for broader rollout.

#### 6.2.4 Phase 4: Scale‐up

The next step is to integrate the selected FemTech solutions into existing health systems and information platforms. This phase involves expanding workforce development, securing ongoing funding, and ensuring interoperability as the digital solutions are introduced to new regions. Active engagement from policymakers, health system managers, and funders is essential to achieve successful scale‐up and long‐term sustainability.

#### 6.2.5 Phase 5: Sustainability and maintenance

The final step focuses on ensuring the long‐term sustainability of the interventions. It involves setting up ongoing training programs, providing regular software updates, maintaining devices, and securing a reliable supply chain for consumables. A monitoring and evaluation system should be established, with dashboards that track indicators across several domains: (1) access and coverage (e.g., antenatal care visit rates, proportion of women using FemTech by region and socioeconomic stratum); (2) process quality (e.g., guideline adherence rates, referral timeliness); (3) clinical outcomes (e.g., rates of preterm birth, pre‐eclampsia, and maternal mortality); (4) cost‐effectiveness (e.g., cost per complication averted); and (5) equity (usage by rural/urban location, income, and age).

## 7 CONCLUSION

Maternal and perinatal mortality and morbidity rates in LMICs remain unacceptably high due to limited quality care, weak systems, and sociocultural and financial barriers. FemTech can enhance access, support providers, empower women, and make pregnancy care safer and more equitable across LMICs. However, its uptake in these countries remains limited by infrastructure, regulatory, affordability, and sociocultural issues. In this context, FIGO is committed to providing practical, global guidance on implementing FemTech in LMICs. This document is the first step toward this goal.

In the current document, we outlined a practical framework to guide implementation. This stepwise approach can help countries move beyond pilots and adopt selected digital solutions in a safe, equitable, and sustainable manner. Still, it is important to remember that technology alone cannot close the gap between LMICs and HICs, and that digital tools can only support and strengthen core health system functions, not replace them.

If thoughtfully designed, FemTech can evolve from a collection of isolated digital tools into an integrated ecosystem that supports continuous maternal care. Mobile connectivity, real‐time symptom reporting, AI‐supported triage, and natural language guidance may allow pregnant women to actively participate in monitoring their health, recognizing danger signs earlier, and engaging their families in preventive behaviors and actions.

Looking ahead, additional evidence is needed to support and guide the implementation of FemTech in LMICs. Head‐to‐head trials comparing FemTech‐enabled care pathways with standard care are needed to demonstrate the clinical benefits and, more importantly, the cost‐effectiveness of digital interventions in LMICs. In parallel, consensus on standardized indicators for monitoring implementation success is urgently needed to enable cross‐country learning and support the transition from isolated pilots to sustained, scalable programs.

## AUTHOR CONTRIBUTIONS

HD, NM, and MH: substantial contributions to the conception or design of the work, drafting the work. MCU, FMM, LF, HS, GJ, VS, EMVDB, NP, GS, VB, MCH, JK, BB, AC, MO, and AP: reviewing the manuscript critically for important intellectual content. All authors: agreement to be accountable for all aspects of the work in ensuring that questions related to the accuracy or integrity of any part of the work are appropriately investigated and resolved.

## CONFLICT OF INTEREST STATEMENT

The authors have no conflicts of interest.

## Data Availability

Data sharing is not applicable to this article as no new data were created or analyzed in this study.
